# Maternal Nutrition during Pregnancy Affects Testicular and Bone Development, Glucose Metabolism and Response to Overnutrition in Weaned Horses Up to Two Years

**DOI:** 10.1371/journal.pone.0169295

**Published:** 2017-01-12

**Authors:** Morgane Robles, Camille Gautier, Luis Mendoza, Pauline Peugnet, Cédric Dubois, Michèle Dahirel, Jean-Philippe Lejeune, Isabelle Caudron, Isabelle Guenon, Sylvaine Camous, Anne Tarrade, Laurence Wimel, Didier Serteyn, Hélène Bouraima-Lelong, Pascale Chavatte-Palmer

**Affiliations:** 1 UMR BDR, INRA, ENVA, Université Paris Saclay, Jouy en Josas, France; 2 Normandie Univ, UNICAEN, EA2608, OeReCa, USC-INRA, Caen, France; 3 Clinique Equine, Faculté de Médecine Vétérinaire, Université de Liège, Liège, Belgium; 4 IFCE, Station Expérimentale de la Valade, Chamberet, France; Medical University of Vienna, AUSTRIA

## Abstract

**Introduction:**

Pregnant mares and post-weaning foals are often fed concentrates rich in soluble carbohydrates, together with forage. Recent studies suggest that the use of concentrates is linked to alterations of metabolism and the development of osteochondrosis in foals. The aim of this study was to determine if broodmare diet during gestation affects metabolism, osteoarticular status and growth of yearlings overfed from 20 to 24 months of age and/or sexual maturity in prepubertal colts.

**Material and methods:**

Twenty-four saddlebred mares were fed forage only (n = 12, group F) or cracked barley and forage (n = 12, group B) from mid-gestation until foaling. Colts were gelded at 12 months of age. Between 20 and 24 months of age, all yearlings were overfed (+140% of requirements) using an automatic concentrate feeder. Offspring were monitored for growth between 6 and 24 months of age, glucose homeostasis was evaluated *via* modified frequently sampled intra veinous glucose tolerance test (FSIGT) at 19 and 24 months of age and osteoarticular status was investigated using radiographic examinations at 24 months of age. The structure and function of testicles from prepubertal colts were analyzed using stereology and RT-qPCR.

**Results:**

Post-weaning weight growth was not different between groups. Testicular maturation was delayed in F colts compared to B colts at 12 months of age. From 19 months of age, the cannon bone was wider in B *vs* F yearlings. F yearlings were more insulin resistant at 19 months compared to B yearlings but B yearlings were affected more severely by overnutrition with reduced insulin sensitivity. The osteoarticular status at 24 months of age was not different between groups.

**Conclusion:**

In conclusion, nutritional management of the pregnant broodmare and the growing foal may affect sexual maturity of colts and the metabolism of foals until 24 months of age. These effects may be deleterious for reproductive and sportive performances in older horses.

## Introduction

Since the early nineties, many epidemiological and experimental studies have shown that modifications of the nutritional environment during gestation and in childhood can affect offspring health at adulthood. The Developmental Origins of Health and Disease (DOHaD) were first demonstrated in human populations [1], with low birthweight neonates or *in utero* underfed infants during the Dutch Hunger Winter having a higher risk of developing a metabolic syndrome as adults [2,3]. Effects of maternal nutritional imbalance on offspring health were subsequently confirmed using animal models [4].

Studies on DOHaD remain scarce in horses [5]. Embryo transfers between breeds of different size were performed to enhance or reduce fetal growth. Postnatal growth patterns were affected from birth until 3 years of age. Moreover, cardiovascular function, glucose homeostasis and endocrine function were altered until weaning [6–12]. Despite the fact that severe maternal undernutrition at mid-gestation related to an infection with *Streptococcus equi* was shown in one study to induce intra-uterine growth retardation (IUGR) [13], most other studies on effects of maternal nutrition indicate that moderate maternal nutritional excess or undernutrition in gestation does not affect birthweight [14–18]. Nevertheless, excess maternal nutrition during pregnancy, with or without carbohydrate supplementation, can affect glucose and lipid metabolism in foals until 160 days of age [13,15,17]. Moreover, an epidemiological study performed in Belgium in a large cohort of horses demonstrated that the incidence of osteochondrosis (OC) is higher in foals born to dams fed concentrates during gestation compared to foals whose dams received forage only [19].

Although there is limited evidence in humans and other species that maternal nutrition may affect offspring reproductive function [20], to our knowledge, consequences on sperm production and fertility remain unknown [21]. Of particular interest for the present study, maternal gestational undernutrition in rats and sheep affects pituitary responsiveness to Gonadotropin Releasing Hormon (GnRH) and testicular development and delays the onset of puberty [22–25]. Also, excess maternal nutrition in domestic animals has been shown to affect testicular development and to delay the onset of puberty [26–28]. To date, these effects have not been studied in horses.

In the equine industry, it is common practice to feed pregnant mares and post-weaning foals with concentrates in order to enhance the growth of fetuses and then foals that will be sold at 1 or 2 years of age [29,30]. The present study is a follow-up of previous work in which broodmares were supplemented (group B) or not (group F) with concentrates from mid-gestation to term [31]. Briefly, the daily supplementation of B mares with flattened barley (2 to 3 kg) provided additional net energy and digestible proteins from the 7^th^ until the 10^th^ month of gestation compared to F mares, allowing them to maintain optimal body condition throughout pregnancy and lactation. Glycemic and insulinemic peaks were observed after each meal. F mares, fed forage only, were energy deficient and lost body condition until parturition. Lipomobilization was increased (as demonstrated by increased Non Esterified Fatty Acids plasma concentrations (NEFA)) as well as insulin sensitivity during late gestation in F mares, that were thus considered underfed compared to B mares that had maintained body condition. From birth until weaning, no difference was observed in foals’ body measurements nor endocrine function (Insulin-like Growth Factor 1 (IGF-1), triiodothyronine (T3), thyroxine (T4) and Non Esterified Fatty Acids (NEFA)). F foals, however, tended to have decreased glucose tolerance and lower osteocalcin concentrations at 3 days of age. At 6 months of age (weaning), these differences had disappeared and osteoarticular status did not differ between both groups of foals at that stage [31].

Here we compare growth, glucose metabolism, testicular maturation and joint health in these foals and yearlings from weaning to 24 months of age. In this period, a 5 month overnutrition challenge was performed from 20 to 24 months of age.

## Materials and Methods

### Animals

#### Ethical statement

The animal studies were approved by the local animal care and use committee (“Comité des Utilisateurs de la Station Expérimentale de Chamberet”) and received ethical approval from the local ethics committee (« Comité Régional d’Ethique pour l’Expérimentation Animale du Limousin ») under protocol number 5-2013-5.

#### Experimental design, management and feeding of mares and foals

**Fig 1:**
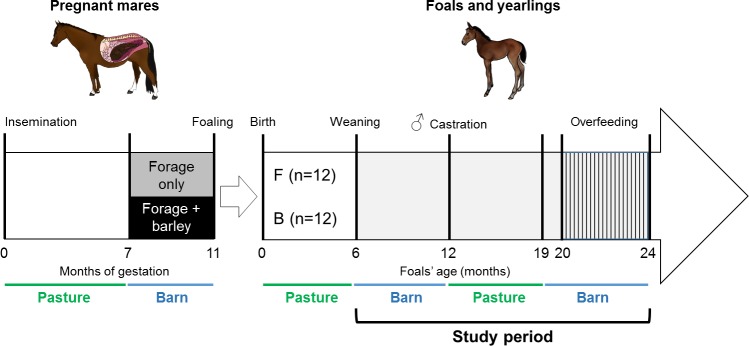
Experimental design from insemination of the broodmares until foals’ 24 months of age. From insemination until 7 months of gestation, pregnant mares were managed as one same herd in the same pasture. From the 7^th^ month of gestation, they were housed in individual boxes and allocated to one of two groups: fed with forage only (F, n = 12) or with forage and flattened barley (B, n = 12) until foaling. From 3 days until 6 months of age, foals were in pasture with their dams [31]. They were weaned at 6 months and housed in open barns and fed the same amount of feed, following the INRA recommendations for growing foals [29]. Colts were castrated at 12 months as a routine procedure. From 12 to 19 months of age, all yearlings were managed in the same pasture. At 19 months of age, all yearlings were housed in open barns and between 20 and 24 months of age, they were over nourished using an automatic feeder (+140% of INRA energy requirements, +135% of NRC energy requirements hatched part).

The experimental design is presented in Fig 1. Twenty-four (n = 2 primiparous and n = 22 multiparous) saddlebred mares (mean age 10 years; range 5–21 years) were fed forage only (F, n = 12) or cracked barley and forage (B, n = 12) from the 7^th^ month of gestation until foaling. Management of mares and foals from insemination to weaning has been described previously by Peugnet *et al*. [31].

In the present paper, colts born to primiparous dams (1 colt from a F dam and 1 colt from a B dam from the same protocol) have been added in the results analysis.

In their first winter, foals were housed in open barns and fed hay and concentrates in agreement with current INRA recommendations for growing foals [31]. Six months after weaning (April 25^th^, at 11 months of age), yearlings returned to pasture and were managed in one same herd with free access to water and minerals salts. On November 25^th^, at 19 months of age, all animals returned to open barns. Animals were separated in 3 groups sorted by bodyweight in 3 different stalls to reduce competition for food (housing group). They were group-fed hay and haylage twice a day and supplemented individually using an automatic concentrate feeder with homemade pellets containing barley, oat, soybean cake, bran, molasses and vitamins and minerals with free access to water. The automatic concentrate feeder allowed yearlings to ingest concentrates at least 4 times a day. The diet was adjusted to meet the requirements during 18 days of adaptation period [29].

From December 13^th^, at 20 months of age, all yearlings were fed to reach an average daily weight gain of 500 g for the upcoming 4 months. Thus, during this period, yearlings were fed 140% of INRA requirements [29] and 135% of the NRC requirements [32], with 62% of the energy of the diet provided by the pellets and 38% provided by forages. A different haylage batch was used at the end of the feeding challenge because the batch used as the start was out of stock. Table 1 presents the daily nutritional supply, Table 2 the quality of feedstuff (INRA feeding system) expressed as net energy (Horse Feed Unit–HFU–; 1 HFU = 2250 kcal), proteins (Horse Digestible Crude Proteins, HDCP), fibers (Raw Cellulose, RC), calcium and phosphorus and Table 3 the quality of feedstuff (NRC feeding system) expressed as digestible energy (estimated using the percentage of difference between net energy and digestible energy for the feedstuff, Mcal) and crude proteins (analyzed) during this period. Concentrate intake was monitored by the automatic concentrate feeder. As forage was distributed as bulk, however, individual forage intake and refusals were not monitored.

All animals were vaccinated and dewormed as for standard care.

**Table 1:** Daily nutritional supply given to yearlings during feeding challenge (19 to 24 months).

Median age (days)	Median age (months)	Type of Haylage	Hay (kg)	Haylage (kg)	Homemade pellets (kg)
**576**	19	H1	4	2.5	2
**583**	20	H1	4	2.5	4
**594**	20	H1	4	2.5	6
**698**	23	H2	4	2.5	6

H1 and H2 indicate when a different hay batch was used. N = 24.

**Table 2:** quality of feedstuff from INRA [29] given to yearlings during feeding challenge (19 to 24 months).

		Chemical composition (per kg of dry matter)			Mineral composition (per kg of dry matter)	
	Dry matter (%)	Net energy (Horse feed units, 1 HFU = 2250 kcal)	Horse digestible crude proteins (g)	Raw cellulose (g)	Calcium (g)	Phosphorus (g)
**Homemade pellets**	88.8	1.13	102	63.9	7.10	4.90
**Hay**	87.3	0.55	75	320.2	4.51	1.92
**Haylage 1**	78.8	0.72	90.58	239.1	9.44	2.29
**Haylage 2**	76.9	0.69	50.06	225.8	6.25	2.96

**Table 3:** Quality of feedstuff from NRC [32] given to yearlings during feeding challenge (19 to 24 months).

	Chemical composition (per kg of dry matter)	
	Estimated digestible energy (Mcal)	Crude proteins (g)
**Homemade pellets**	3.55	126.3
**Hay**	1.60	60.1
**Haylage 1**	1.95	160.1
**Haylage 2**	1.86	111.1

#### Body measurements

At 6, 12, 19 and 24 months of age, foals and yearlings were weighed and withers’ height and cannon width were measured. Body condition score (BCS, scale 1 to 5 [33]) was assessed by two independent trained operators at 19 and 24 months of age.

### Evaluation of glucose metabolism

#### Fasting blood glucose

At 12 months of age, yearlings were fasted overnight with free access to water before blood sampling in the morning by jugular puncture into 10-ml EDTA coated tubes. Glucose blood concentration was immediately analyzed using a glucometer (Freestyle optium, Abbott, USA). Plasma was separated after 10 min centrifugation at 5500 rpm and stored at -20°C.

#### Modified frequently sampled intravenous glucose tolerance test (FSIGT)

Modified FSIGT were performed at 19 (before nutritional challenge) and 24 months of age (at the end of the feeding challenge). The FSIGT is a method that enables the simultaneous evaluation of insulin sensitivity, glucose tolerance and insulin secretion by the pancreas [34]. Animals had free access to hay and water during the test. Both jugular veins were catheterized (Introcan, BBraun, Germany) 30 min before the beginning of the test. One catheter was used for infusion and the other one for sampling. Glucose (0.30 g/kg) was injected over 2 min (19 months of age) and 5 min (24 months of age) and 10 ml samples were collected on EDTA coated tubes at -5 min and 5, 7, 13, and 19 min after the injection started. At 20 min, a diluted solution of 30 mUI/kg insulin (Umuline Eli Lilly, USA) in isotonic sodium chloride (Aguettant, France) was injected over 1 min. Blood collection was performed 2, 6, 15, 25, 40, 70, 100, 130 and 160 min after insulin injection. Blood samples were processed as for fasting samples. FSIGT were not performed on two yearlings of each group due to behavioral issues.

#### Insulin assays

Plasma insulin concentrations were measured using a human insulin AlphaLISA immunoassay kit (PerkinElmer, USA).

Before the assay, inactivated and insulin-free fetal bovine serum (FBS) was prepared. FBS was heated at 55°C for 1 hour and filtered on a 0.22-μm membrane. Insulin was removed using 50 mg/ml of active charcoal at 4°C for 30 min. Three 20-min centrifugations at 4000 rpm were used to remove the charcoal and the FBS was filtered again on a 0.22 μm membrane and kept in aliquots at -20°C. Standard samples of decreasing insulin concentrations were prepared in inactivated and insulin-free fetal bovine serum (FBS).

Plasma samples were thawed and maintained at 4°C during the time of assay. Four microliter samples were distributed in duplicate in opaque 96 well plates (½ AreaPlate^TM^, PerkinElmer, USA). A 4-μL mix of anti-insulin antibody (1 nM final concentration) and acceptor-beads (10 μg/ml final concentration) was then added. After 1 hour incubation at room temperature in the dark, 32 μL of streptavidin-coated donor beads (40 μg/ml final concentration) were added. Plates were analyzed after 30 min incubation in the dark at room temperature, using the Enspire® reader and Manager software (PerkinElmer, USA).

The minimum level of detection was 5.3 mUI/L. Intra- and inter-assay coefficients of variation were 6% and 7%, respectively. The assay was validated by dilutional parallelism between standard curve and endogenous insulin and expected values against obtained values.

#### Calculation of glucose homeostasis parameters from modified FSIGT assays

**Fig 2:**
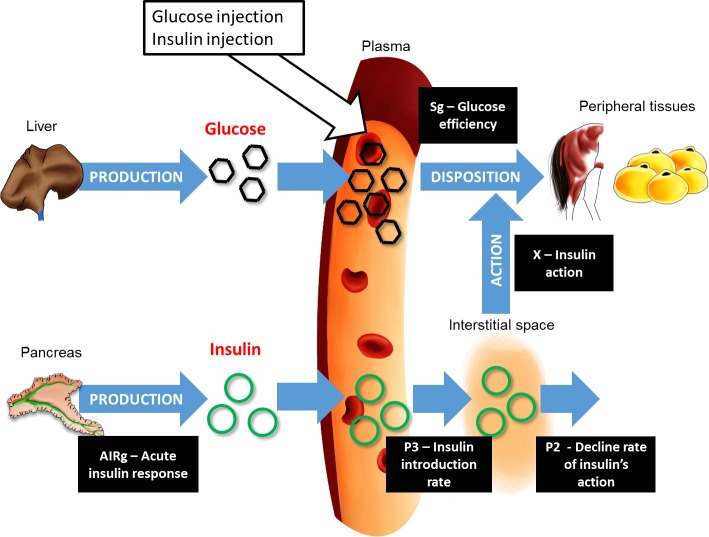
Schematic representation of the Bergman’s minimal model (adapted from [34–36,38]). Glucose is produced and released by the liver in a basal state. After a meal, or a glucose intra-venous injection, the levels of plasma glucose increase. From the blood, the glucose will be disposed in the peripheral tissues by two ways:
By itself. The glucose efficiency (**Sg**) is the capacity of the glucose to mediate its own disposal.Mediated by insulin. The elevated concentration of blood glucose induces the production of insulin by the pancreas. The acute insulin response (**AIRg**) represents the production of insulin by the pancreas during the first 10 minutes after glucose injection. Insulin is transferred into the interstitial space (**P3—insulin introduction rate**) from the bloodstream and reaches the peripheral tissues to mediate glucose disposal (**X -insulin action**). With time, insulin action declines at a rate calculated by the **P2** proxy. **Insulin sensitivity** (**IS**) is calculated as P2/P3. Finally, the disposition index (**DI**) (multiplication of IS and AIRg) is used to describe β-cells responsiveness.

Glucose effectiveness (Sg), acute insulin response to glucose (AIRg), insulin sensitivity (IS) and the disposition index (DI) were calculated using the Bergman’s minimal model [34–36] on the MinMod Millennium software (Ver 6.02, MINMOD Inc., 2001) [37]. Schematic representation of the different proxies calculated *via* the minimal model is presented in Fig 2.

### Testicles

#### Tissue sampling and treatment

Testicles from prepubertal stallions (group F: n = 6 and group B: n = 7) aged 43–50 weeks were collected during routine castration. Castration were performed in order to have no significant difference in the age of the animals at the time of castration between groups. One sample was snap frozen on dry ice and stored at -80°C for mRNA extraction while the other sample was fixed in 4% Paraformaldehyde (PFA)/ Phosphate Buffered Saline (PBS) for at least 24 hours, dehydrated in a graded ethanol series, paraffin embedded and sectioned (5 μm) for stereological analysis and immunohistochemistry. Samples were randomly chosen.

#### Morphologic evaluation

To analyze testicular morphology, 5-μm sections were stained with 1% hematoxylin, 1% eosin and 0.2% green light solution using a classic protocol. Pictures were acquired with ScanScope® CS (Aperio, Leica). Testicular maturation was assessed with a stereological point-counting frame for whole slide image [39] on Stereology Toolkit (ADCIS S.A, Saint-Contest, France) using ImageScope (Leica Biosystems). A minimum of 202 points per grid was applied to each image with a 500-μm interval between points. The first point was placed randomly. Seminiferous tubules were classified according to maturation stage in prepubertal testis based on the lumen score (LS) system [40]. Three non-serial sections per animal (F group: n = 6 and B group: n = 7) were examined.

#### Immunohistochemistry

Sections were deparaffinized and rehydrated in graded ethanol series. Slides were then treated with 3% H_2_O_2_ in PBS for 10 min to inactivate endogenous peroxidase activity. Sections were boiled for 20 min in 0.1 M citrate buffer (pH 6.1) at 450 W in a microwave. Slides were blocked with PBS/triton 0.2%/dry milk 1% for 1 hour before overnight incubation with the primary antibodies directed against Proliferating Cell Nuclear Antigen (PCNA) (FL-261, sc-7907, SantaCruz, USA) diluted 1:50 in PBS/0.5% dry milk in a moist chamber at 4°C. After washing in PBS-Triton 0.2%, sections were incubated for 1h30 at room temperature with biotin-labeled goat anti-rabbit IgG and streptavidin-HRP conjugate secondary antibody (1:500) (A16096, ThermoScientific) and rinsed, followed by DAB (3,3′N-diaminobenzidine, R&D Systems) incubation. Sections were counterstained with hematoxylin and mounted in Eukitt® medium (SigmaAldrich, USA). For negative control, the primary antibody was omitted. Analyses were performed with Olympus AX70 microscope and analyzed by AnalySIS getIT (Olympus). Six non-serial sections per animal (4 animals/group) were examined to estimate PCNA immunolabelling.

#### Quantitative real time PCR

Total mRNA was extracted with Tri-Reagent kit (Sigma-Aldrich, France) according to the manufacturer’s instructions. Total RNA (250 ng) was used for reverse transcription with 100 IU Moloney murine leukemia virus reverse transcriptase (Promega, France), 20 IU RNasin (Promega, France), 500 μM dNTPs (Promega, France) in a 20 μl final volume for 90 min at 37°C. Real time PCR was performed with 5 μl of diluted cDNA (1:10), 0.5 μM of each primer (Eurogentec, France) and 10 μl of GoTaq qPCR MasterMix2X (Promega, France) in a 20 μl final volume. PCR reactions were performed in a Stratagene Mx3005P apparatus (Agilent, France). Negative control contained water and all set primers were designed to be placed on different exons. The relative gene expression was normalized with GAPDH RNA expression.

The genes analyzed were markers of testicular maturity (Cyp19, Connexin 43 (Cx43), Androgen Receptor (AR), Steroidogenic Acute Regulatory Protein (STAR)), markers of immaturity (Anti-müllerian hormone (AMH)) and genes involved in the establishment of the blood-testis barrier (Occludin, N cadherin). Primer sequences are presented in Table 4.

**Table 4:** Primers sequences used for amplification of equine products in RT-qPCR assays in testicles of prepubertal colts.

Gene	GenBank accession number	Primer	Size of PCR product (pb)
***Anti-müllerian hormone (AMH)***	JF330269,1	Forward: 5'—CCATCTGGAGGAGCCAAC—3'	80
		Reverse: 3'—CCCGTGACAGTGACCTCAG—5'	
***Androgen Receptor (AR)***	NM_001163891,1	Forward: 5'—AGTACTCCTGGATGGGGCTT—3'	132
		Reverse: 3'—TGTACATCCGGGACTTGTGC—5'	
***B Catenin***	NM_001122762,1	Forward: 5' -ACAATGGCTACTCAAGCCGA- 3'	78
		Reverse: 3'—CCAGTGACTAACAGCCGCTT- 5'	
***Connexin 43 (Cx43)***	XM_005596901,1	Forward: 5'—CTCTTCACCAACCGCTCCT—3'	68
		Reverse: 3'—CTGTCTCCGGTAACCAGCTT—5'	
***Cyp 19***	NM_001081805,1	Forward: 5' -AAAGCCACCCGGTTCCTAAC- 3'	119
		Reverse: 3' -CCTTGATCTCAGGCGAAGCA- 5'	
***Glyceraldehyde 3-phosphate dehydrogenase (GAPDH)***	AF157626	Forward: 5' -CCAGAACATCATCCCTGCTT—3'	158
		Reverse: 3' -CGTATTTGGCAGCTTTCTCC- 5'	
***N Cadherin***	XM_001495782,3	Forward: 5' -GCCATTCAGACTGACCCGAA- 3'	100
		Reverse: 3'—CTGCAGCGACAGTAAGGACA- 5'	
***Occludin***	XM_001504044,3	Forward: 5' -AAGCTTCCATGTCAGTGCCTT- 3'	84
		Reverse: 3' -TTGCTTGGTGCGTAATGATTGG—5'	
***p450c17***	D88184,1	Forward: 5'—CTGGGAGCAGGACAGAACAG- 3'	126
		Reverse: 3' -CTGGGAGCAGGACAGAACAG- 5'	
***Steroidogenic Acute Regulatory Protein (STAR)***	NM_001081800,1	Forward: 5'—TGGCCTATATCCAGCAGGGA- 3'	142
		Reverse: 3' -AATACCTTGCCCACGTCTGG- 5'	
***Tight Junction Protein 1 (TJP1)***	XM_005602842,1	Forward: 5' -CCATTGCCCTCCCAGTACAC—3'	148
		Reverse: 3' -GAGGTGGGTCTGGTTTGGAC- 5'	
***3beta-Hydroxysteroid dehydrogenase (3βHSD)***	NM_001081911,1	Forward: 5'—CTTGCTGGTGGAGGAGAAGG- 3'	106
		Reverse: 3' -CAGCTTGACCTTGCTCTGGA- 5'	

### Radiographic evaluation of osteoarticular status in yearlings

Yearlings were sedated using butorphanol (Dolorex®, 0.02 mg/kg IV, Intervet, France) combined with romifidine (Sedivet®, 0.06 mg/kg IV, Intervet, France) for radiographic examination at 24 months of age. Examinations were performed as previously reported [19] but development was made with a Vetray CR 2430 scanner (Sedecal, Spain). Radiographs were examined with an Examion CR Vita 45 (Carestream Health, USA) and analyzed blindly by two experienced examiners in a digital format with the Vita CR System Software V.3.2. (Carestream Health, USA). OC was diagnosed according to the presence of lesions that were previously described [19,41,42]. Yearlings were classified as OC-positive according to the presence of one or more OC lesions identified by both examiners. One yearling of each group was not examined because of technical issues.

### Statistical analysis

Results are expressed as median [quartile 1 –quartile 3] and presented as curves (median and interquartile range IQR) or boxplots (minimum to maximum). Statistical analyses were performed using the R software, version 3.0.2 [43]. Homoscedasticity and normality were tested before performing the statistical analyses.

There was no effect of sex of the foal nor of the interaction sex of the foal:diet of the dam for any measurements. These factors were thus removed from statistical models.

The longitudinal data of nutritional intake, growth and metabolism were analyzed using a type III ANOVA on a mixed linear model taking into account the dam's diet, time and interaction diet:time as fixed effects and the individual as random effect. For growth, a cubic B spline function was applied on the size at birth to include divergences of growth between yearlings. To analyze BCS and metabolism between 19 and 24 months of age, the housing group was added as fixed effect. When global differences were observed, t-test was used to compare groups at each time-point.

Testicular data were analyzed using a multiple factor analysis (MFA) [44] of the FactoMineR package [45]. The MFA is a factorial method that enables the study of several groups of variables defined on the same set of individuals. Gene expression results were considered as one group of variables, stereological results as the other. Individuals and variables were graphically represented on the two first dimensions and a clustering analysis was applied on the data to confirm the results observed on the graphs. The age and weight of the foal at gelding did not affect any of the results and were therefore not included in the final analysis.

Radiographic results were analyzed using a chi-squared test.

Effects were considered significant when p-values<0.05.

In total, 24 foals (F, n = 12; B, n = 12) were obtained from weaning. All the foals and yearlings were measured from weaning until 24 months of age. FSIGT was performed on 20 foals and yearlings (F, n = 10; B, n = 10) due to behavioral issues. Analyses of testicles were performed on 13 colts (F, n = 6; B, n = 7). Radiography examinations were performed on 22 yearlings (F, n = 11; B, n = 11) due to technical issues (Table 5).

**Table 5:** Summary of the number of foals included in each analysis.

Analyses	Number of F foals	Number of B foals	Sex of foals	Reason
**Feeding**	12	12	M & F	
**Body measurements**	12	12	M & F	
**FSIGT**	10	10	M & F	Behavioral issues
**Testicles analyses**	6	7	M	
**Radiography examination**	11	11	M & F	Technical issues

## Results

### Nutrition during feeding challenge from 19 to 24 months of age

**Fig 3:**
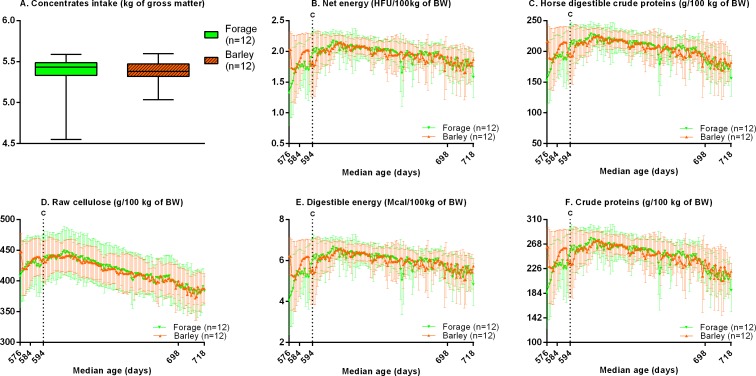
**Daily feed intake (median and IQR) in terms of kg of brute matter of concentrates (A), net energy (B), estimated digestible energy (C), horse digestible crude proteins (D), crude proteins (E) and raw cellulose (F) during the feeding challenge of overnutrition.** HFU: horse feed units, BW: Bodyweight, C: Beginning of the feeding challenge.

Energy (p = 0.9), proteins (p = 0.9), fibers (p>0.9), calcium (p>0.9), phosphorus (p = 0.8) and concentrate intake (p = 0.7) did not differ between groups (Fig 3).

### Growth and body condition

#### Growth

**Fig 4:**
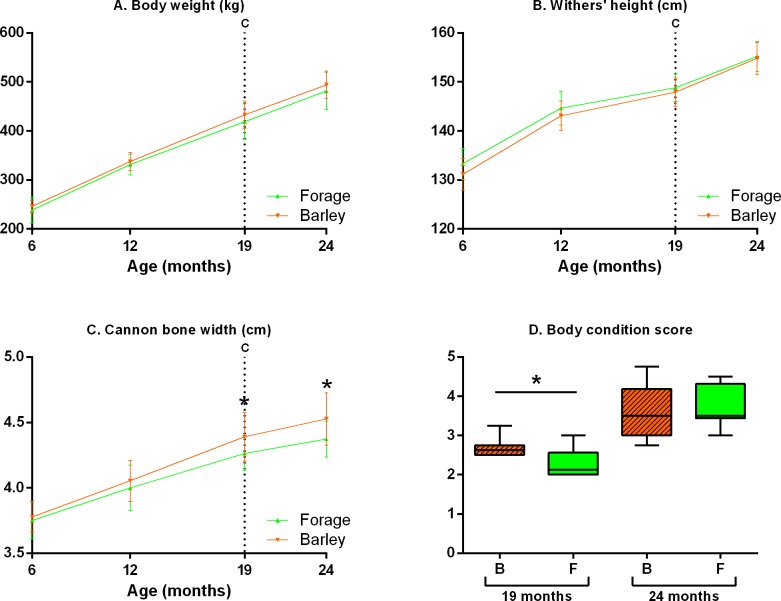
**Foals’ and yearlings’ (median and IQR) body weight (A), withers’ height (B) and cannon bone width (C) between 6 and 24 months of age and body condition score (D) at 19 and 24 months of age.** B: Foals born to dams fed with barley and forage (n = 12), F: Foals born to dams fed with forage only (n = 12), C: Beginning of the feeding challenge. Values marked with an asterisk are significantly different (p<0.05).

Diet of the dam did not affect foals’ and yearlings’ weight (p = 0.3) nor withers height (p = 0.2) at any time. The cannon bone, however, was narrower in F *versus* B yearlings at 19 (p = 0.02) and 24 months (p = 0.01) of age. During the feeding challenge, median daily weight gain was 476 g [364–517]. Average daily gain did not differ between groups at any time (p = 0.1) (Fig 4A, 4B and 4C). Yearlings of both groups had the same growth between 6 and 24 months.

#### Body condition

F yearlings were leaner than B yearlings by 0.5 point of BCS at 19 months of age (p = 0.02, Fig 4D). At 24 months this difference had disappeared (p = 0.7). BCS gain during the challenge was not different between groups (p = 0.1). F yearlings had slightly less fat than B yearlings before overnutrition but this difference disappeared after.

### Glucose metabolism

Data from glucose metabolism analyses are presented in Table 6.

**Table 6:** Effects of the feeding challenge and age (median and IQR) on acute insulin response to glucose (AIRg), insulin sensitivity (IS), disposition index (DI) and glucose effectiveness (Sg), basal plasma glucose (GB) and basal plasma insulin (IB) in yearlings whose dams were fed with barley and forage (B, n = 10) or forage only (F, n = 10), at 19 and 24 months of age.

Age (month)	19		24	
Diet of dam	B	F	B	F
**AIRg mU.min.L^-1^**	22.3^a^ [17.0–47.1]	46.0^ab^ [35.2–74.1]	74.1^bc^ [37.1–108.3]	109.9^c^ [81.7–142.6]
**IS x10^-4^ L.mU^-1^.min^-1^**	3.3^a^ [1.6–4.7]	1.8^b^ [1.4–2.2]	1.3^b^ [0.6–3.0]	1.7^b^ [1.1–2.3]
**DI x10^-2^**	75.4^a^ [62.2–79.7]	95.3^ab^ [48.0–152.9]	115.2^ab^ [37.4–335.1]	158.2^b^ [96.5–238.6]
**Sg x10^-2^.min^-1^**	0.010 [0.008–0.016]	0.008 [0.006–0.009]	0.013 [0.010–0.015]	0.010 [0.007–0.012]
**GB mg.dL^-1^**	114^a^ [108.5–124.9]	122.5^a^ [116–131.4]	79.5^b^ [73.9–85.6]	93.5^c^ [89.6–94.4]
**IB mU.L^-1^**	4.3^a^ [3.5–5.4]	5.9^ab^ [3.7–6.7]	9.6^b^ [6.0–10.2]	8.2^b^ [6.0–10.6]

Different letters correspond to statistically different results (p<0.05), the use of the same letter corresponds to statistically non-different results (p>0.05).

#### Fasting and basal plasma glucose and insulin

Fasting glucose and insulin plasma concentrations were not affected by the dam's diet at 12 months of age (p>0.9 and p = 0.3, respectively).

The nutritional challenge (from 19 to 24 months) enhanced basal insulin plasma concentrations in B but not F group (p<0.01 and p = 0.5, respectively). Basal glycemia did not differ at 19 months of age (p = 0.2) but was higher in F *versus* B yearlings at 24 months of age (p = 0.02). Moreover, all yearlings had a lower basal glycemia at 24 compared to 19 months of age (p<0.0001). In addition, basal plasma glucose/insulin ratio (<0.0001) was lower in the B group at 24 months compared to 19 months of age. These results indicate alterations of metabolism of F foals towards insulin resistance at 24 months, as they were hyperglycemic compared to B foals.

#### FSIGT at 19 and 24 months of age

**Effect of maternal nutrition.** At 19 and 24 months, there was no difference for acute response of the pancreas, (AIRg, p = 0.4, p = 0.5, respectively), disposition index (DI, p = 0.8, p = 0.4, respectively) and Glucose effectiveness (Sg, p = 0.1, p>0.9, respectively) between groups. Sensitivity to insulin (IS), however, was lower in group F *versus* group B at 19 months but not at 24 months of age (p = 0.048 and p = 0.9, respectively). F yearlings were more insulin resistant than B yearlings at 19 months of age.

**Effect of the feeding challenge.** After the end of the feeding challenge, F and B yearlings had higher pancreas acute response to glucose injection (AIRg, p = 0.02, p<0.01) than before the feeding challenge. At 24 months of age, insulin sensitivity, IS, was reduced in group B (p = 0.05) without changes in group F (p = 0.9) compared to 19 months of age. At 24 months, glucose effectiveness (Sg) remained unchanged in both groups (p = 0.5 in F and p = 0.5 in B). These data indicate that overnutrition decreased IS and increased acute production of insulin by the pancreas in B yearlings. In F yearlings, the feeding challenge increased the pancreas acute response only. Overnutrition affected more the carbohydrate metabolism of B yearlings than F yearlings.

### Testes

**Fig 5:**
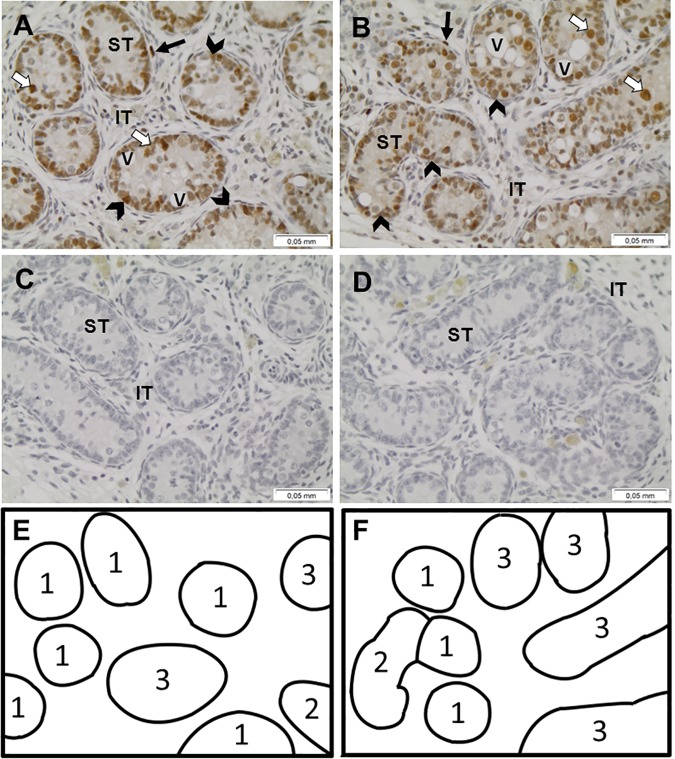
Immunohistochemical detection of PCNA protein in equine prepubertal testes. Representative testicular sections for foals born to forage fed (A, n = 6), or barley and forage fed dams (B, n = 7), with respective negative controls (C and D). Lumen scores are represented within each tubule for F foals in testicular section (E) and B foals in testicular section (F). Arrow heads indicate Sertoli cells, white arrows, gonocytes and black arrows, peritubular cells. ST: seminiferous tubule, IT: interstitial tissue, V: vacuoles. All cellular types constituting the seminiferous epithelium, Sertoli cells and gonocytes are positive for PCNA staining. Scale bar = 0.05 mm.

The proliferative activity of Sertoli cells was analyzed by PCNA immunohistochemistry. A strong immunostaining was visible in the basal region of the seminiferous epithelium associated both with Sertoli cells and gonocytes for both groups. Immunolabelling was also observed in peritubular cells (Fig 5).

The morphologic evaluation of testicular sections was realized according to the lumen score system reported by Heninger [40]. Due to the foals’ age, only immature stages (LS 1 to 4) were observed: LS 1) tubules without a lumen; LS 2) tubules containing a single vacuole; LS 3) tubules containing several independent vacuoles and LS 4) tubules containing several aggregating vacuoles. LS 1 was the most represented in each group (group B: 43% and group F: 46%).

**Fig 6:**
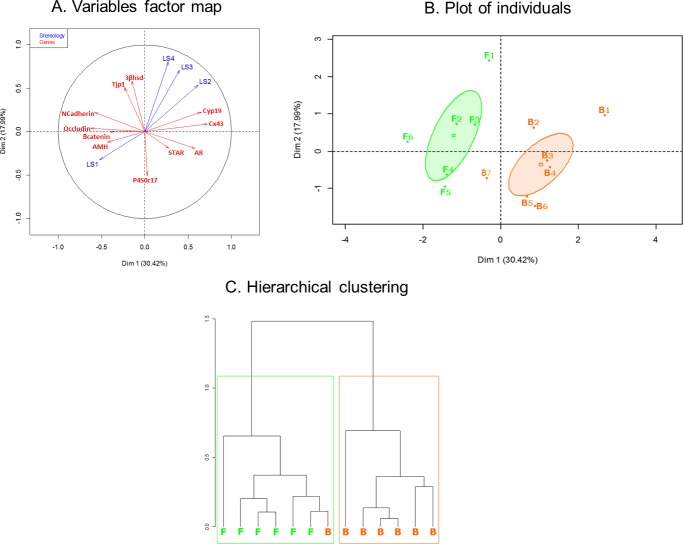
**Variables factor map (A), plot of individuals (B) and hierarchical clustering (C) of the Multiple Factor Analysis (MFA) applied on gene expression (red arrows) and stereological (blue arrows) results.** On the plot of individuals, foals born to dams that were fed with forage only (n = 6) are represented by the letter F and foals born to dams that were fed with barley and forage (n = 7) are represented by the letter B.

Functional and structural data obtained on the testicles by RT-qPCR and stereology were analyzed by MFA. On the variables factor map (Fig 6A), the first dimension (30.42% of the variability) separated the markers of testicular maturity. The expression of genes used as markers of immaturity (AMH) and involved in the establishment of the blood-testis barrier (Occludin, N cadherin) is located on the left side together with the first lumen score. The expression of genes indicating advanced testicular maturity (Cyp19, Cx 43, AR, STAR) is located on the right side together with the most advanced lumen scores. Group F clustered on the left of the plot whereas group B clustered on the right of the plot, with one B foal clustering with the F foals, very close to the middle of the plot, as shown Fig 6B. This was further confirmed by the hierarchical clustering analysis (Fig 6C).

In conclusion, testicles of F foals were less mature than those of B foals at 12 months of age.

### Osteoarticular status

Seven animals were OC-positive at 24 months of age: 4 yearlings (2 fillies and 2 colts) in the F group (36.4%) *versus* 3 yearlings (2 colts and 1 filly) in the B group (27.3%). The proportion of yearlings with lesions did not differ neither between groups (p>0.9) nor between fillies and colts (p>0.9). Three of the F yearlings that had OC had only one lesion, localized either in the left hock, right stifle or left fetlock, whereas the last F yearling had lesions in both left and right hocks. In the group B, the 3 yearlings had only one lesion, either in the left fetlock, the left or the right hock. F and B yearlings were affected equally with OC lesions.

## Discussion

In the present study, growth of foals from 6 to 24 months of age was not affected by maternal diet. Maternal undernutrition, however, seemed to affect bone growth as F foals had narrower cannons than B foals from 19 months of age but there was no difference for OC lesions between both groups of yearlings at 24 months of age. Moreover, testicles of F yearlings were less mature than those of the B group at 12 months of age, indicating that maternal undernutrition affected prepubertal testicular maturation. The metabolism of foals was also disturbed by maternal undernutrition: at 19 months of age, F yearlings had decreased insulin sensitivity and increased basal glycaemia compared to B yearlings. From 19 to 24 months of age, all yearlings were overfed up to approximately 140% and 135% of INRA and NRC energy requirements, respectively [29,32] and ingested the same amount of concentrate throughout the feeding challenge. Although BCS gain was not affected, the feeding challenge enhanced acute pancreatic response and a decreased basal glycemia after overnutrition in both groups. Overfeeding affected B yearlings more strongly than F yearlings, as decreased insulin sensitivity and enhanced basal insulinemia were observed in B, but not F yearlings.

To our knowledge, there is no other reported study in horses evaluating the effect of overfeeding young horses born to dams fed different amounts and quality of energy sources during gestation. Studies in other species (sheep, goats and rabbits) indicate that maternal undernutrition increases offspring spontaneous feed intake, especially, but not only, when IUGR is observed [46–48], possibly due to effects on late gestational of early neonatal plasma leptin concentrations as leptin is necessary for the maturation of hypothalamic pathways leading to food intake regulation [48–51]. In the present study, we did not observe any difference in foal birthweight [31]. Consistent with our results, maternal undernutrition in late gestation in rabbits and sheep, with or without birthweight reduction, did not affect food intake in offspring whether or not they were submitted to an overnutrition challenge [52,53]. Nevertheless, in a study where goats were underfed in late pregnancy, effects on food intake were observed only in adult offspring [48,54]. Therefore, the yearlings studied here may have been yet too young to express these effects [54].

Before the feeding challenge, at 19 months of age, F yearlings were less insulin sensitive than B yearlings. Already at 3 days of age, F foals tended to be less glucose tolerant than B foals, which suggests that this insulin resistance persisted during growth [31]. In humans, epidemiological data from the Dutch Hunger Winter show that children exposed to maternal undernutrition in late gestation tend to have increased risks of developing type 2 diabetes at adulthood [55]. In sheep, offspring born to dams underfed in late gestation were shown to be glucose intolerant at 1 year of age, although, in that study, birth weight and post-natal growth did not differ from controls [56]. In the horse, pancreatic maturation takes place near term (290–327 days of gestation) [57]. In the present study, maternal undernutrition occurred from 200 to 321 days of gestation [31] when the foal’s pancreas was maturing. The relative insulin resistance of F foals and yearlings might be an effect of a default in pancreatic maturation induced by maternal undernutrition during fetal development. As all foals were fed with concentrates during their first winter (6–11 months), it could be speculated that the difference between pre- and post-natal nutrition may have affected F foals more than B foals as indicated by the Predictive Adaptive Response hypothesis [58].

After the feeding challenge, these differences disappeared but basal glycemia was higher in the F group than in the B group. Consistent with these results, saddlebred foals that suffered IUGR through embryo transfer in ponies had higher fasting glycemia until 1.5 year of age [10,11].

The overfeeding challenge reduced insulin sensitivity in the B group only. The pancreas responded to overfeeding by secreting more insulin (increased AIRg), leading to higher basal insulin and lower basal glucose plasma concentrations. Consistently, in the adult horse, a diet rich in starch decreases insulin sensitivity, inducing an enhanced AIRg with or without an increase in basal insulin concentrations [38,59,60]. In contrast, the overfeeding challenge did not affect insulin sensitivity in F yearlings. Nevertheless, the pancreatic response to glucose, AIRg, using FSIGT test was higher at 24 months than at 19 months of age. Thus the increased pancreatic insulin secretion in response to the carbohydrate-enriched diet seemed to overcome the lower insulin sensitivity and led to a decreased basal glycemia in both groups of animals at the end of the challenge. We hypothesize that F yearlings were less affected than B yearlings because they were already insulin resistant before the beginning of the feeding challenge. as shown in sheep, where overnutrition affected more the carbohydrate metabolism of lambs born to overnourrished compared to undernourrished dams in late gestation [61].

In sheep, late maternal undernutrition did not affect fat deposition in offspring, although a decreased subcutaneous:mesenteric fat ratio was observed [53]. In contrast, others showed that sheep underfed during gestation have a higher fat mass than controls [56,62]. In most studies, however, fat composition is measured through *post-mortem* weighing of central and peripheral adipose tissues or through alternative methods (such as deuterium oxide infusion, impedancemetry or densitometry) whereas in the present study only the overall peripheral adipose quantity was semi-quantitatively assessed through BCS. Interactions between glucose metabolism and fat deposition can therefore not be inferred from this study.

From weaning to the beginning of the feeding challenge, growth was not affected by maternal diet. B yearlings, however, had larger cannon bones than F yearlings at 19 and 24 months of age. In horses, the cannon bone width is linked to housing and exercise: foals housed in pastures or trained extensively have larger cannon bones than foals housed in stalls or with no exercise [63,64]. Increases in cannon bone circumference have also been associated to increased bone strength and mineral contents [63,64]. Here, both groups were housed in the exact same environment starting from birth but their exercise was not monitored. It has been shown in the rabbit and the rat that maternal undernutrition during pregnancy led to sedentary behavior in offspring [47,65]. Therefore, it could be possible that F foals were less active than foals from B group. This modification of the locomotor behavior could explain the difference of cannon width between both groups of foals.

As OC status is stable from 18 months of age [66], the feeding challenge applied to the yearlings in the present study did not affect the OC results. The correlation between cannon bone width and OC lesions has been rarely tested in the literature and the results are inconsistent [67,68]. Unlike what was shown epidemiologically [19], the maternal diet in the present study did not affect the development of OC lesions neither at 6 months of age—where there was only a tendency for an increase in OC lesions in B foals [31]—nor at 24 months of age. It has been shown, however, that feeding yearlings 4 times/day decreases both the postprandial glycemic peak and the area under the curve of the glycemia throughout the day compared to yearlings receiving 2 meals/day [69], suggesting that postprandial insulin concentrations may also have been decreased in the present study as yearlings were fed at least 4 times/day between 6 and 12 months of age. Since OC in foals has been linked to postprandial insulin plasma concentrations [70], we hypothesize that the use of an automatic concentrate feeder from 6 to 12 months of age in the present study may have reduced postprandial insulin levels compared to animals fed twice daily in a more traditional way and thus reduced the incidence of OC [71].

Puberty in stallions begins around 12 months and ends around 2 years of age [72,73]. Testes were collected at a pre-pubertal stage (10 to 12 months of age). At this stage, the seminiferous epithelium is composed of proliferative Sertoli cells, gonocytes and/or spermatogonia. In equine testes, the markers of puberty initiation were determined from observations that the acquisition of mature phenotype for Sertoli cells is associated with loss of AMH [74] and with an increase in junctional proteins (cx 43) [40,75]. In contrast, steroidogenic activity and sensibility to androgen increase with testicular maturation [75,76]. The effects observed here on testicular development, could be due to a direct action occurring during fetal life. Indeed, the change in maternal diet occurred at the beginning of testicular inguinal migration in fetuses (about 270 days of gestation) [77], when testicular differentiation is still regulated. Indeed, in rats and sheep, maternal undernutrition reduced seminiferous tubule diameter and Sertoli cell numbers per tubule [24,25,78]. Another hypothesis would have been an effect of hormones: T3, leptin, insulin and IGF-1 plasma concentrations are known to increase with improved nutritional status in the fetus and neonate [31,79]. These hormones are directly active on testes [80,81]. Insulin and IGF-1 regulate Sertoli cell proliferation and FSH action in mice [80] and T3 affects Sertoli cell differentiation and blood-testis barrier assembly [81]. At 6 months of age, however, no differences were found in insulin levels and glucose metabolism, T3, and IGF-1 levels in both groups of foals [31], but there is no information available on earlier stages.

## Conclusions

In conclusion, we showed that broodmare management in gestation may affect testicular maturation of colts at 12 months of age, glucose homeostasis of post-weaning yearlings, body condition score and cannon bone width from 19 months of age. Excess nutrition in the second winter after birth also affected the metabolism of B and F yearlings in a different manner, with no difference in growth pattern. Nevertheless, the study was performed on a limited number of animals of the same breed (Anglo-Arabian) and further confirmation on a larger number of animals and/or in another breed remains mandatory.

## Supporting Information

S1 Fig Validations of insulin assay. A. Dilutional parallelism between standard curve and endogenous insulin. To linearize the 4PL curve, logit was calculated as logit = log ((AlphaLISA signal (count)–minimum asymptote) / (maximum asymptote–AlphaLISA signal (count))). B. Expected insulin values against obtained values. Linear regression statistic test was applied to compare the equality of slope to 1 and intercepts to 0. Run-test was performed to determine whether data deviated significantly from the linear model. For both tests and for all samples, p<0.5. C. Bland-Altman graph comparing the expected insulin values against the obtained values.
(TIF)

S2 Fig Effects of feeding challenge and age (median and IQR) on glucose and insulin during the modified frequent sampling intra venous glucose tolerance test in yearlings whose dams were fed with barley and forage (B, n = 10) or forage only (F, n = 10), at 19 and 24 months of age. (TIF)

S3 Fig Comparison of growth rate between the weight of foals and yearlings measured during the experimentation and the expected weight calculated using the 1–3 equation of NRC 2007 [32]. B: Foals born to dams fed with barley and forage (n = 12), F: Foals born to dams fed with forage only (n = 12). Values marked with an asterisk are significantly different (p<0.05).
(TIF)

S1 Table Composition of the vitamin and minerals complement given to foals and yearlings in pasture. (DOCX)

S2 Table Composition of concentrate supplement given to foals and yearlings in open barns. (DOCX)

S3 Table Detailed calculations to obtain the DE (NRC 2007 [32]) values for feed (A) and comparison between calculated DE and NRC recommendations for growing foals (B). A. Estimated DE has been calculated using the equation DE (Mcal) = (HFU*2.25)/ % value; “% value” being the % of difference between HFU and DE for a same feed of the same dry matter content. B. Comparison of calculated DE values with NRC recommendations for growing foals between 20 and 24 months calculated using the equation DE (Mcal/d) = ((56.5*X*10^−0.145^)*BW)+((1.99+1.21*X-0.021*X^2^)*ADG). Where X = age in months, ADG = average daily gain in kg and BW = body weight in kg.
(DOCX)

S4 Table Comparison of growth rate between the weight of foals and yearlings measured during the experimentation and the expected weight calculated using the 1–3 equation of NRC 2007 [32] and the estimated mature weight of the animals by averaging the weight of the sire and the weight of the dam at insemination. A. Calculation of expected weight and difference between calculated weight and real weight for each foal and yearling between 6 and 24 months. B. For animals of each group (B and F) at 6, 12, 19 and 24 months, median of real weight and calculated weight and results of statistical analyses (Type III Anova, mixed linear model, fixed effect: Group, random effect: Individual). C. For animals of each group (B and F), at 6, 12, 19 and 24 months, median of difference between calculated weight and real weight and results of statistical analysis.
(DOCX)
