# Supplementary figures and images for: Maternal Nutrition during Pregnancy Affects Testicular and Bone Development, Glucose Metabolism and Response to Overnutrition in Weaned Horses Up to Two Years
Source: PLoS One. 2017 Jan 12;12(1):e0169295. doi: 10.1371/journal.pone.0169295 (PMC5231272; doi:10.1371/journal.pone.0169295)

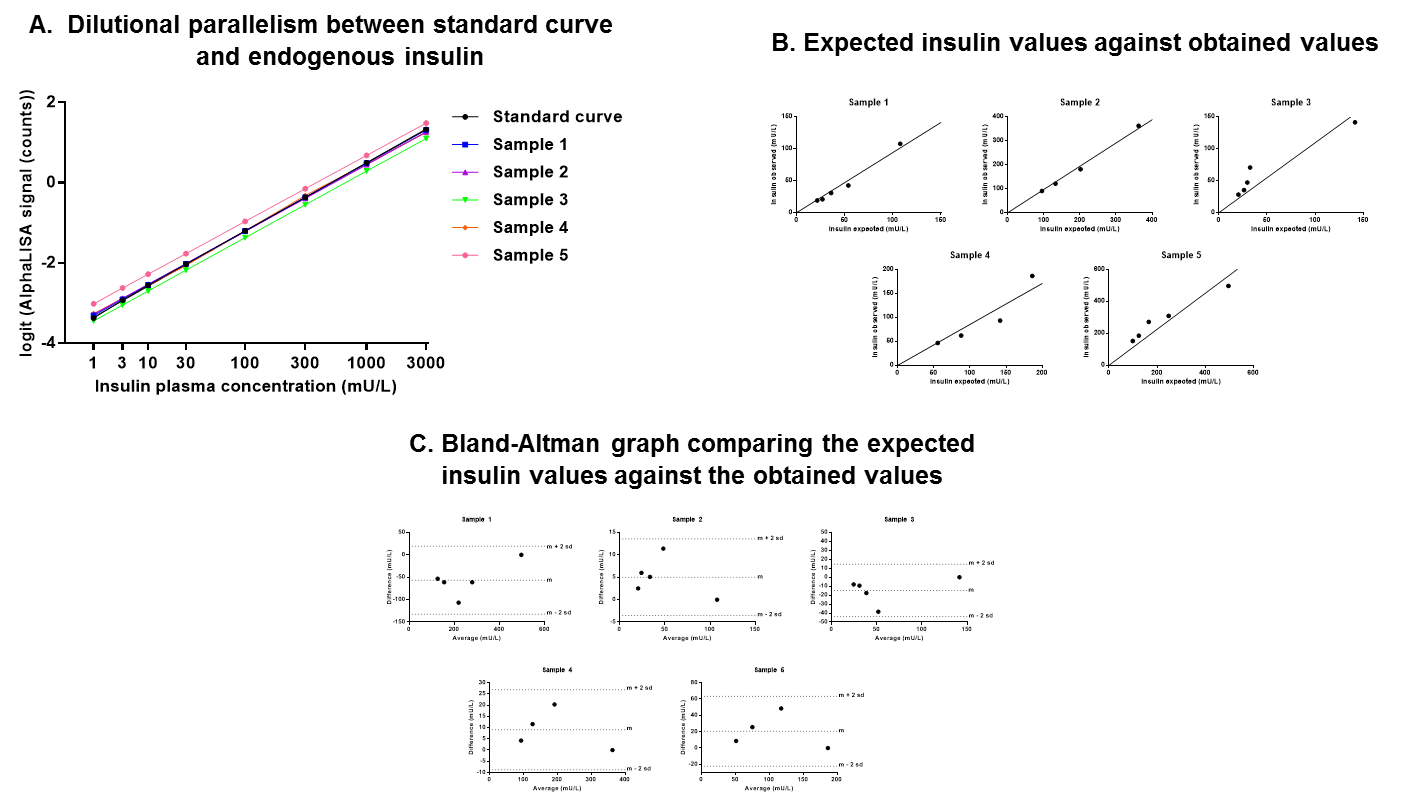

Supplement: S1 Fig — A. Dilutional parallelism between standard curve and endogenous insulin. To linearize the 4PL curve, logit was calculated as logit = log ((AlphaLISA signal (count)–minimum asymptote) / (maximum asymptote–AlphaLISA signal (count))). B. Expected insulin values against obtained values. Linear regression statistic test was applied to compare the equality of slope to 1 and intercepts to 0. Run-test was performed to determine whether data deviated significantly from the linear model. For both tests and for all samples, p<0.5. C. Bland-Altman graph comparing the expected insulin values against the obtained values. (TIF) [file pone.0169295.s001.tif]
