# Supplementary material for: Maternal Nutrition during Pregnancy Affects Testicular and Bone Development, Glucose Metabolism and Response to Overnutrition in Weaned Horses Up to Two Years
Source: PLoS One. 2017 Jan 12;12(1):e0169295. doi: 10.1371/journal.pone.0169295 (PMC5231272; doi:10.1371/journal.pone.0169295)
Supplement: S1 Table — (DOCX) [file pone.0169295.s004.docx]

| **Composition** | Calcium carbonate, monocalcium phosphate, magnesium oxide, lithothamne, cane molasses, sodium chloride, magnesium phosphate, plants |
| --- | --- |
| **Vitamins / kg of brute matter** | |
| Vitamin A | 300 000 IU |
| Vitamin D3 | 90 000 IU |
| Vitamin E | 1000 IU |
| **Micronutrients / kg of brute matter** | |
| Copper sulfate pentahydrate | 570 mg |
| Copper chelate of glycine hydrate | 30 mg |
| Zinc oxide | 3560 mg |
| Zinc chelate of glycine hydrate | 190 mg |
| Manganese oxide | 3000 mg |
| Anhydrous calcium iodate | 55 mg |
| Cobalt basic carbonate monohydrate | 25 mg |
| Sodium selenite | 18 mg |
