# Supplementary material for: Maternal Nutrition during Pregnancy Affects Testicular and Bone Development, Glucose Metabolism and Response to Overnutrition in Weaned Horses Up to Two Years
Source: PLoS One. 2017 Jan 12;12(1):e0169295. doi: 10.1371/journal.pone.0169295 (PMC5231272; doi:10.1371/journal.pone.0169295)
Supplement: S2 Table — (DOCX) [file pone.0169295.s005.docx]

| **Composition** | Barley, oat, soybean cake, bran, cane molasses, calcium carbonate, dicalcium phosphate, sodium chloride, magnesium oxide, sodium sulfate |
| --- | --- |
| **Vitamins / kg of brute matter** | |
| Vitamin A | 10 400 IU |
| Vitamin D3 | 2300 IU |
| Vitamin E | 10 IU |
| **Micronutrients / kg of brute matter** | |
| Copper sulfate pentahydrate | 30 mg |
| Ferrous carbonate | 15 mg |
| Zinc oxide | 179 mg |
| Manganese oxide | 120 mg |
| Anhydrous calcium iodate | 1.3 mg |
| Cobalt basic carbonate monohydrate | 0.40 mg |
| Sodium selenite | 0.1 mg |
