# Supplementary material for: Maternal Nutrition during Pregnancy Affects Testicular and Bone Development, Glucose Metabolism and Response to Overnutrition in Weaned Horses Up to Two Years
Source: PLoS One. 2017 Jan 12;12(1):e0169295. doi: 10.1371/journal.pone.0169295 (PMC5231272; doi:10.1371/journal.pone.0169295)
Supplement: S3 Table — Detailed calculations to obtain the DE (NRC 2007 [32]) values for feed (A) and comparison between calculated DE and NRC recommendations for growing foals (B). A. Estimated DE has been calculated using the equation DE (Mcal) = (HFU*2.25)/ % value; “% value” being the % of difference between HFU and DE for a same feed of the same dry matter content. B. Comparison of calculated DE values with NRC recommendations for growing foals between 20 and 24 months calculated using the equation DE (Mcal/d) = ((56.5*X*10−0.145)*BW)+((1.99+1.21*X-0.021*X2)*ADG). Where X = age in months, ADG = average daily gain in kg and BW = body weight in kg. (DOCX) [file pone.0169295.s006.docx]

|  | **Feed name** | **DM % as fed** | **NE (HFU/kg)^3^** | **DE (Mcal/kg)^2^** | **% value** | **% value used** |
| --- | --- | --- | --- | --- | --- | --- |
| **Concentrate** | Barley, grain, rolled | 86.7 | 1.14 | 3.85 | 0.70 | 0.716 |
|  | Oat, grain, rolled | 88.1 | 0.99 | 3.21 | 0.69 |  |
|  | Soybean, meal | 80.0 | 0.94 | 3.16 | 0.70 |  |
|  | Wheat bran | 86.6 | 0.89 | 3.13 | 0.72 |  |
|  | Molasses, beet sugar | 73.0 | 0.88 | 3.80 | 0.77 |  |
| **Haylage** | Mostly grass silage, mature | 55.0 | 0.47 | 2.87 | 0.84 | 0.836 |
| **Hay** | Mostly grass hay, immature | 85.0 | 0.54 | 2.37 | 0.77 | 0.772 |

A

B

| **Yearling** | **Age (months)** | **Body weight (kg)** | **ADG (kg/d)** | **Theoretical calculated DE (Mcal/d)^2^** | **Mean ingested DE (Mcal/100kgBW/d)** | **Difference (%)** |
| --- | --- | --- | --- | --- | --- | --- |
| 1 | 20.3 | 426.5 | 0.63 | 26.9 | 27.5 | 2.3 |
| 2 | 20.2 | 492.5 | 0.47 | 26.3 | 28.0 | 6.1 |
| 3 | 20.2 | 408.2 | 0.46 | 23.2 | 27.6 | 16.1 |
| 4 | 19.7 | 451.6 | 0.61 | 27.3 | 27.6 | 1.4 |
| 5 | 19.7 | 425.4 | 0.67 | 27.5 | 26.2 | 4.8 |
| 6 | 19.8 | 461.3 | 0.47 | 25.3 | 26.8 | 5.5 |
| 7 | 19.3 | 424.8 | 0.57 | 25.6 | 27.3 | 6.4 |
| 8 | 18.9 | 412.9 | 0.78 | 28.7 | 27.4 | 4.4 |
| 9 | 18.9 | 399.8 | 0.52 | 23.8 | 28.2 | 15.6 |
| 10 | 18.4 | 455.9 | 0.66 | 28.3 | 27.7 | 1.9 |
| 11 | 20.1 | 453.6 | 0.61 | 27.5 | 27.5 | 0.2 |
| 12 | 20.1 | 385.2 | 0.64 | 25.5 | 27.9 | 8.5 |
| 13 | 19.9 | 411.9 | 0.77 | 28.8 | 27.7 | 3.8 |
| 14 | 19.7 | 460.4 | 0.69 | 29.2 | 27.6 | 5.5 |
| 15 | 19.7 | 496.9 | 0.63 | 29.3 | 27.0 | 7.7 |
| 16 | 19.6 | 415.3 | 0.52 | 24.4 | 24.4 | 0.3 |
| 17 | 19.6 | 401.1 | 0.43 | 22.3 | 27.3 | 18.1 |
| 18 | 19.5 | 410.3 | 0.65 | 26.5 | 28.2 | 6.0 |
| 19 | 19.1 | 445.6 | 0.25 | 20.8 | 28.0 | 25.6 |
| 20 | 19.1 | 390.7 | 0.56 | 24.1 | 27.9 | 13.4 |
| 21 | 19.5 | 383.8 | 0.64 | 25.4 | 27.7 | 8.5 |
| 22 | 18.7 | 369.8 | 0.61 | 24.3 | 28.5 | 14.7 |
| 23 | 18.9 | 401.8 | 0.64 | 25.9 | 28.5 | 9.0 |
| 24 | 19.4 | 446.1 | 0.57 | 25.2 | 27.5 | 8.6 |
