# Supplementary material for: Maternal Nutrition during Pregnancy Affects Testicular and Bone Development, Glucose Metabolism and Response to Overnutrition in Weaned Horses Up to Two Years
Source: PLoS One. 2017 Jan 12;12(1):e0169295. doi: 10.1371/journal.pone.0169295 (PMC5231272; doi:10.1371/journal.pone.0169295)
Supplement: S4 Table — A. Calculation of expected weight and difference between calculated weight and real weight for each foal and yearling between 6 and 24 months. B. For animals of each group (B and F) at 6, 12, 19 and 24 months, median of real weight and calculated weight and results of statistical analyses (Type III Anova, mixed linear model, fixed effect: Group, random effect: Individual). C. For animals of each group (B and F), at 6, 12, 19 and 24 months, median of difference between calculated weight and real weight and results of statistical analysis. (DOCX) [file pone.0169295.s007.docx]

| **A** | | | **Real weight (kg)** | | | | **Calculated weight (kg)** | | | | **Difference between calculated weight and real weight (kg)** | |
| --- | --- | --- | --- | --- | --- | --- | --- | --- | --- | --- | --- | --- |
| **Yearling** | **Group** | **Estimated mature weight (kg)** | **6 months** | **12 months** | **19 months** | **24 months** | **6 months** | **12 months** | **19 months** | **24 months** | **12 months** | **19 months** |
| **1** | **B** | 604.41 | 241.0 | 332.1 | 412.9 | 510.0 | 261.11 | 388.03 | 478.70 | 518.59 | 55.93 | 65.80 |
| **2** | **B** | 575.56 | 252.6 | 334.2 | 412.4 | 483.1 | 248.64 | 369.51 | 455.85 | 493.83 | 35.31 | 43.49 |
| **3** | **B** | 588.59 | 250.0 | 336.4 | 419.9 | 485.1 | 254.27 | 377.87 | 466.16 | 505.01 | 41.47 | 46.26 |
| **4** | **B** | 593.11 | 246.0 | 365.6 | 446.8 | 518.9 | 256.23 | 380.78 | 469.75 | 508.89 | 15.18 | 22.95 |
| **5** | **B** | 608.11 | 252.8 | 361.9 | 455.9 | 523.9 | 262.70 | 390.40 | 481.62 | 521.75 | 28.50 | 25.72 |
| **6** | **B** | 611.04 | 265.0 | 361.0 | 472.3 | 544.3 | 263.97 | 392.29 | 483.94 | 524.27 | 31.29 | 11.62 |
| **7** | **B** | 574.76 | 234.0 | 315.6 | 399.8 | 474.0 | 248.30 | 369.00 | 455.21 | 493.15 | 53.40 | 55.41 |
| **8** | **B** | 579.74 | 252.2 | 343.5 | 454.4 | 513.9 | 250.45 | 372.19 | 459.15 | 497.42 | 28.69 | 4.75 |
| **9** | **B** | 539.76 | 216.0 | 315.0 | 395.4 | 459.4 | 233.17 | 346.52 | 427.49 | 463.11 | 31.52 | 32.12 |
| **10** | **B** | 569.49 | 249.6 | 327.7 | 414.9 | 495.6 | 246.02 | 365.61 | 451.04 | 488.62 | 37.91 | 36.14 |
| **11** | **B** | 584.99 | 247.0 | 318.8 | 440.5 | 515.3 | 252.72 | 375.56 | 463.31 | 501.92 | 56.76 | 22.81 |
| **12** | **B** | 529.84 | NA | 285.7 | 401.8 | 438.0 | 228.89 | 340.16 | 419.63 | 454.60 | 54.46 | 17.83 |
| **13** | **F** | 584.36 | 259.0 | 331.9 | 447.1 | 500.3 | 252.45 | 375.16 | 462.82 | 501.38 | 43.26 | 15.72 |
| **14** | **F** | 574.89 | 251.4 | 346.8 | 459.2 | 543.6 | 248.35 | 369.08 | 455.31 | 493.26 | 22.28 | -3.89 |
| **15** | **F** | 608.39 | 271.8 | 370.5 | 487.6 | 583.8 | 262.82 | 390.59 | 481.84 | 522.00 | 20.09 | -5.76 |
| **16** | **F** | 565.24 | 231.6 | 319.6 | 377.4 | 456.5 | 244.18 | 362.88 | 447.67 | 484.98 | 43.28 | 70.27 |
| **17** | **F** | 596.49 | 226.4 | 326.0 | 379.9 | 462.5 | 257.68 | 382.95 | 472.42 | 511.79 | 56.95 | 92.52 |
| **18** | **F** | 550.91 | 217.2 | 303.3 | 411.2 | 497.7 | 237.99 | 353.69 | 436.32 | 472.68 | 50.39 | 25.12 |
| **19** | **F** | 568.31 | 266.0 | 344.4 | 447.4 | 521.8 | 245.51 | 364.86 | 450.10 | 487.61 | 20.46 | 2.70 |
| **20** | **F** | 552.19 | 188.0 | 297.6 | 388.4 | 490.3 | 238.55 | 354.51 | 437.33 | 473.78 | 56.91 | 48.93 |
| **21** | **F** | 567.44 | 215.4 | 329.9 | 405.9 | 482.0 | 245.13 | 364.30 | 449.41 | 486.86 | 34.40 | 43.51 |
| **22** | **F** | 566.09 | 227.0 | 322.2 | 399.0 | 497.8 | 244.55 | 363.43 | 448.34 | 485.70 | 41.23 | 49.34 |
| **23** | **F** | 546.24 | NA | 300.4 | 369.8 | 472.7 | 235.98 | 350.69 | 432.62 | 468.67 | 50.29 | 62.82 |
| **24** | **F** | 537.91 | 268.0 | 353.7 | 397.9 | 464.0 | 232.38 | 345.34 | 426.03 | 461.53 | -8.36 | 28.13 |
|  |  |  |  |  |  |  |  |  |  |  |  |  |
|  | **Percentage of mature weight calculated using the 1-3 equation of the NRC 2007²** | | | | | | **0.432** | **0.642** | **0.792** | **0.858** |  |  |

**B**

| **Age (months)** | **6** | **12** | **19** | **24** |
| --- | --- | --- | --- | --- |
| **B median real weight (kg)** | 249.6 | 333.2 | 417.4 | 502.8 |
| **F median real weight (kg)** | 231.6 | 328.0 | 402.5 | 494.0 |
| **B median calculated weight (kg)** | 251.6 | 373.9 | 461.2 | 499.7 |
| **F median calculated weight (kg)** | 244.8 | 363.9 | 448.9 | 486.3 |
| **B pvalue (calculated weight *vs* real weight)** | 0.38 | <0.0001 | <0.01 | 0.9 |
| **F pvalue (calculated weight *vs* real weight)** | 0.21 | <0.0001 | <0.01 | 0.16 |
| **C** |  |  |  |  |
| **Age (months)** | **6** | **12** | **19** | **24** |
| **B median calculated weight - real weight** | 249.6 | 333.2 | 417.4 | 502.8 |
| **F median calculated weight - real weight** | 231.6 | 328.0 | 402.5 | 494.0 |
| **Pvalue** | 0.86 | 0.63 | 0.72 | 0.28 |
